# SARS-CoV-2 with Influenza B Coinfection in a Patient with Sickle Cell HbSC Presenting with Painful Crisis: A Case Report

**DOI:** 10.7759/cureus.56102

**Published:** 2024-03-13

**Authors:** Elrazi A Ali, Abdalla Fadul, Eihab A Subahi, Mugtaba Ahmed, Ahmed Elamin, Malar Thwin, Edouard Guillaume

**Affiliations:** 1 Internal Medicine, Interfaith Medical Center, Brooklyn, USA; 2 Internal Medicine, Hamad Medical Corporation, Doha, QAT; 3 Hematology and Medical Oncology, Interfaith Medical Center, Brooklyn, USA

**Keywords:** covid-19, sars-cov-2 infection, influenza b, adult sickle cell anemia, sickle cell hbsc

## Abstract

Sickle cell disease is a hereditary red blood cell disorder characterized by hemolytic anemia, particularly in association with stress. As they grow, most children with sickle cell anemia undergo auto-splenectomy, making them vulnerable to serious infections. Patients with sickle cell disease infected with the SARS-CoV-2 virus are reported to have an increased risk for hospitalization, thrombosis, and other complications compared to non-sickle cell patients. Influenza infection in patients with sickle cell is associated with increased morbidity. Patients with sickle cell HbSC are reported to have a milder form of the disease than HbSS. Coinfection with SARS-CoV-2 and influenza B is rarely reported in patients with hematologic diseases, including sickle cell hemoglobinopathy. We are reporting an unusual case of a patient with sickle cell HbSC with co-infection of SARS-CoV-2 and influenza B with a favorable outcome.

## Introduction

SARS-CoV-2 is the causative virus for coronavirus disease 2019 (COVID-19). Since the first pandemic wave, the disease has had various presentations. Common symptoms include shortness of breath, cough, fever, and fatigue. Almost all major organs can be affected by a SARS-CoV-2 virus infection, including the lungs, skin, liver, kidneys, brain, hematopoietic system, and pancreas [1-3]. The interaction between the virus and the immune system is more complex, particularly after the emerging mutations resulting in variants that vary in virulence. Furthermore, the different available vaccines have boosted the immunological response against the virus. Patients with comorbid conditions and elderly are more likely to develop more serious infections [4,5]. Patients with hematologic malignancies also might have severe disease [6]. Influenza infection in sickle cell disease is associated with increased morbidity [7]. Co-infection of influenza virus and SARS-CoV-2 virus is rarely reported in sickle cell patients. We are reporting an interesting SARS-CoV-2 and influenza B virus co-infection in a sickle cell patient with HbSC.

## Case presentation

**Figure 1:**
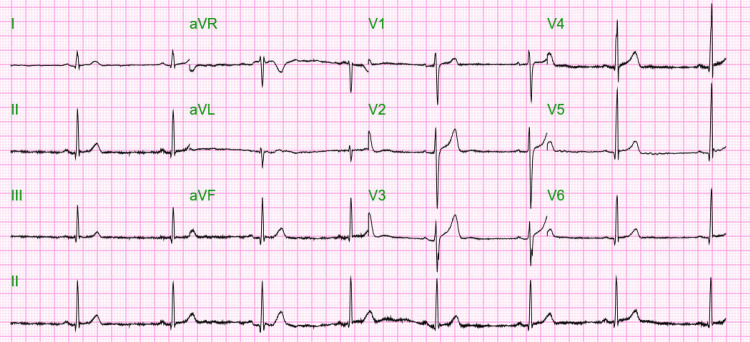
Electrocardiogram (EKG) showing sinus bradycardia

**Figure 2:**
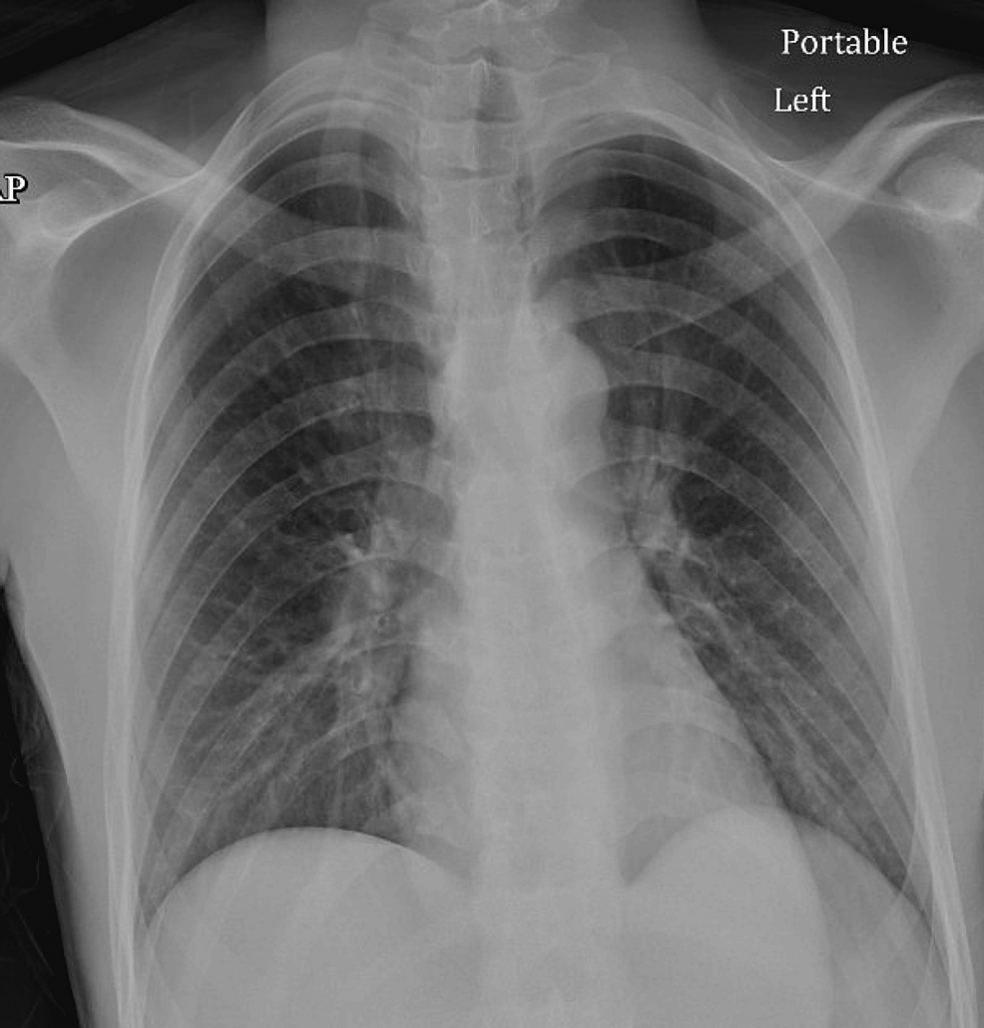
Portable chest x-ray showing no significant infiltrate or consolidation

A 21-year-old male with SC hemoglobinopathy presented to the emergency department with acute vaso-occlusive crisis with the onset of pain in his legs and lower back for two days associated with fever, nonproductive cough, and runny nose. He medicated himself with Tylenol and ibuprofen with no significant relief. No shortness of breath or dizziness was reported in the symptomatology. The patient was vaccinated against COVID-19; received one dose of mRNA vaccine (Pfizer) and did not receive the flu vaccine for this influenza season. His last flu vaccine was in January 2021. Physical examination was unremarkable, blood pressure was 111/73 mmHg, pulse 62 beats per minute, SpO_2_ 97% on room air, body weight 60.8 kg, BMI 17.8. An electrocardiogram showed sinus Bradycardia (Figure 1). Polymerase chain reaction for respiratory viruses was positive for SARS-CoV-2 and influenza type B. Venous blood gas: Ph: 7.34 (normal 7.31-7.41), PO_2_ 45 (30-40 mmHg), PCO_2_ 53 (41-51 mmHg), chest x-ray: was reported negative for infiltrate or consolidation (Figure 2). The patient was started on hydromorphone 4 mg intramuscular and diphenhydramine 25 mg intramuscular every four hours as needed. He received oral hydration, Humidified oxygen with a nasal cannula 2 liter/min, Folic acid, and multivitamins. The patient was started on treatment for COVID-19 with nirmatrelvir/ritonavir (Paxlovid) and Oseltamivir for influenza. The patient was observed for three days, and his symptoms significantly improved, and he was discharged. Blood investigations during the admission are shown in Table 1. On follow-up, he was doing well and had no report of thrombotic events or significant complications.

**Table 1:** Result of blood work done during admission

	Result	Normal range
White blood cell count	11 x10E3/µL	4.5-11 x 10E3/µL
Hemoglobin	11.1 x 10E3/µL	113-117 x 10E3/µL
Platelet count	216 x 10E3/µL	130-400 x 10E3/µL
Absolute lymphocytes	5.2 x 10E3/µL	1-4.8 x 10E3/µL
Absolute neutrophils	4 x 10E3/µL	2-7 x 10E3/µL
Blood urea nitrogen	9 mg/dL	7-25 mg/dL
Creatinine	0.9 mg/dL	0.7-1.3 mg/dL
Sodium	135 meq/L	136-145 meq/L
Potassium	4 meq/L	3.5-5.1 meq/L
Chloride	100 meq/L	98-107 meq/L
HCO3	28 meq/L	21-31 meq/L
Total bilirubin	2.1 mg/dL	0.3-1.0 mg/dL
Albumin	4.1 g/dL	3.5-5.7 g/dL
Total protein	7 g/dL	6.4-8.9 g/dL
international normalized ratio (INR)	1.12	0.85-1.15
Lactate dehydrogenase	274 U/L	140-271 U/L
Ferritin	195.20 ng/mL	23.9-336.2 ng/mL
Hemoglobin A	00%	96.4-98.8%
Hemoglobin A2	3.3%	1.8-3.2%
Hemoglobin S	50%	0%
Hemoglobin C	45%	0%
Hemoglobin F	1.7%	0-2%

## Discussion

Patients with sickle cell disease and sickle cell trait are reported to have an elevated risk of hospitalization and mortality (five times compared to non-sickle cell patients) from SARS-CoV-2 infection [8]. On the other hand, influenza infection in sickle cell patients is associated with increased morbidity [7]. Co-infection of these two viral illnesses is anticipated to have worse outcomes on patients’ health. Respiratory epithelial damage with influenza infection is associated with increased susceptibility to secondary bacterial infection that is associated with poor outcomes [9]. SARS-CoV-2 uses angiotensin-converting enzyme 2 (ACE-2) receptors in the lower airways to enter the cell [10]. Influenza can potentially induce lung injury via ACE-2 receptor-mediated effects [11]. Co-infection with SARS-CoV-2 and influenza B virus is expected to produce a substantial injury due to greater cytokine production, damage to the ciliated epithelium, and a higher risk of superimposed bacterial infection. Our patient was infected with both viruses and had mild symptoms.

Our patient was expected to have a severe disease. However, he developed a milder form. This could be related to various causes. First, viral-related factors: infection with mild strain could result in mild disease. For example, omicron virus infection is reported to cause mild disease in patients with sickle cell anemia [12]; this is not only in sickle cell anemia but also in other hematologic diseases like myeloproliferative neoplasms and chronic lymphocytic leukemia [13-16]. Second, host-related factors, such as the young age and the absence of comorbid medical conditions, are usually associated with lower morbidity and a better outcome. Moreover, prior vaccination is known to provide protection against severe disease from SARS-CoV-2 [6]. Also, sickle cell HbSC is associated with a milder form of anemia compared to sickle cell SS [17].

The data regarding the co-infection of influenza and SARS-CoV-2 infection are scarce [18,19], and there are minimal reports of co-infection in patients with hematologic diseases, including sickle cell disease. Co-infection of the two viruses is not common; a systematic review showed that, on average, 0.8% of patients with confirmed SARS-CoV-2 infections had co-infection with the influenza virus [20], with significant variation depending on the geographical areas of the report. For example, the percentage of patients with COVID-19 who had influenza was 4.5% in Asia and 0.4% in America. This might indicate underreporting of the co-infection in many parts of the world. One research that included intensive care unit (ICU) patients revealed a significant occurrence of co-infection among critical patients, with rates of co-infection reaching 71% in ICU [21]. The increase in reports in critical patients might indicate that co-infection is underreported in patients with mild infection or co-infection is associated with severe disease. Physicians might not test patients with mild disease if the symptoms were explained by one of the two viruses, either SARS-CoV-2 or influenza virus. On the contrary, one study in Mayo showed that co-infection is not associated with worse outcomes; however, most of the studied patients were young and healthy [22]. The major limitation of our case is the sample size; more studies with a larger sample size are needed better to understand the co-infection in patients with sickle cell disease.

Treatment for co-infection is not clear; it is not known which antiviral treatment for ARS-CoV-2 is more effective for patients with concomitant influenza viral infection. Steroids are associated with increased mortality in patients with influenza [23]. However, steroids are associated with opposite effects in patients with ARS-CoV-2 [24]. The different responses to steroids might indicate that the two viruses produce damage in a different way, and their co-infection does not act in a synergistic way. Our patient did not receive steroids and received Oseltamivir and Paxlovid, and he did well. Anti-influenza treatment is not known to affect SARS-CoV-2 or vice versa; large observation studies are required to determine if any medication is more effective than others. Although both antiviral treatments work by different mechanisms, oseltamivir inhibits the activity of the viral neuraminidase enzyme present on the virus's surface, preventing budding from the host cell, viral replication, and infectivity [25]. At the same time, Nirmatrelvir binds directly to the SARS-CoV-2 Mpro active site and suppresses the coronavirus' viral replication process, and ritonavir raises nirmatrelvir plasma levels by reducing its CYP3A-mediated metabolism [26]. In sickle cell patients, hypoxia and respiratory injury can lead to acute vaso-occlusive crisis, as well as acute chest syndrome. Fortunately, these complications did not develop in our patient. Vaccination against both viruses is associated with reduced severity of both infections. The most effective way of preventing severe co-infection is to follow the protective measures and guidelines from the CDC and vaccination to prevent severe illness.

## Conclusions

The interaction between SARS-CoV-2 and the influenza virus is not well understood; some reports show an associated increased mortality among critical patients, while other studies showed mild infection in healthy populations. Due to the similar symptoms and clinical presentation, clinicians must test for both infections to minimize morbidity and mortality associated with coinfection. Studies with larger sample sizes are needed to better understand coinfection-related outcomes and treatments in sickle cell anemia patients.
